# Use and acceptability of an asthma diagnosis clinical decision support system for primary care clinicians: an observational mixed methods study

**DOI:** 10.1038/s41533-024-00401-x

**Published:** 2024-11-27

**Authors:** Luke Daines, Anne Canny, Eddie Donaghy, Victoria Murray, Leo Campbell, Carol Stonham, Heather Milne, David Price, Mark Buchner, Lesley Nelson, Frances S. Mair, Aziz Sheikh, Andrew Bush, Brian McKinstry, Hilary Pinnock

**Affiliations:** 1 Asthma UK Centre for Applied Research, Usher Institute, University of Edinburgh, Edinburgh, UK; 2 NHS Gloucestershire Integrated Care Board, Gloucester, UK; 3 Primary Care Respiratory Society (PCRS), Knowle, UK; 4 South East GP Unit, NHS Education for Scotland, Edinburgh, UK; 5 Observational and Pragmatic Research Institute, Singapore, Singapore; 6 Optimum Patient Care, Cambridge, UK; 7 Centre of Academic Primary Care, Division of Applied Health Sciences, University of Aberdeen, Aberdeen, UK; 8 Tactuum, 280 St Vincent Street, Glasgow, UK; 9 General Practice and Primary Care, School of Health and Wellbeing, College of Medical, Veterinary and Life Sciences, University of Glasgow, Glasgow, UK; 10 Nuffield Department of Primary Care Health Sciences, University of Oxford, Oxford, UK; 11 Imperial Centre for Paediatrics and Child Health and National Heart and Lung Institute, Imperial College, London, UK; 12 Department of Paediatric Respiratory Medicine, Royal Brompton Hospital, London, UK; 13 Centre for Population Health Sciences, Usher Institute, University of Edinburgh, Edinburgh, UK

**Keywords:** Asthma, Diagnosis

## Abstract

There is uncertainty about how best to diagnose asthma, especially in primary care where mis-diagnosis is common. To address this, we developed a clinical decision support system (CDSS) for asthma diagnosis in children and young people (aged 5-25 years). This study explored the feasibility and acceptability of the CDSS in UK primary care. We recruited general practices from England and Scotland. The CDSS was available for use during routine consultations for six months. We analysed CDSS usage and, toward the end of the study, undertook qualitative interviews with clinicians who had used the CDSS. Within the 10 practices who completed the study, the CDSS was used by 75 out of 94 clinicians. 11 clinicians from 8 practices were interviewed. The CDSS was acceptable to participants who particularly commented on the ease of use and auto-population of information from the patient record. Barriers to use included the inability to record findings directly into the patient notes and a sense that, whilst possibly useful for trainees and junior colleagues, the CDSS would not necessarily lead to a change in their own practice. The CDSS was generally well received by primary care clinicians, though participants felt it would be most useful for trainees and less experienced colleagues.

## Introduction

Asthma can be difficult to diagnose, with evidence of misdiagnosis in children and adults^1,2^. Incorrectly labelling someone with asthma can lead to the wrong treatment being given with unwanted side effects and unnecessary financial, social, psychological and environmental cost^3^. Conversely, undiagnosed and untreated asthma risks ongoing symptoms, reduced quality of life, substantial morbidity and even mortality.

Asthma is a heterogenous disease with different underlying disease processes and phenotypes^4^. Typical symptoms of asthma vary over time and intensity and can overlap with other conditions. There is no single test that can confirm or refute an asthma diagnosis in all cases. Instead, diagnostic tests rely on identifying the defining features of asthma, namely variable airflow limitation and/or markers of airway inflammation.

Clinical decision support systems (CDSS) may help to improve the accuracy with which asthma is diagnosed by prompting clinicians to consider a diagnosis, identifying relevant information from patient records, interpreting results of investigations or data from wearable devices, and supporting evidence-based diagnosis, treatment and self-management^5–7^. CDSS could also improve patient involvement in consultations and encourage shared diagnostic decision making by providing information in a patient friendly format^7^.

In previous work, we developed a prediction model to inform primary care clinicians’ assessment of the likelihood of an asthma diagnosis in children and young people aged 5–25 years^8^. Following input from individuals with asthma, parents^9^ and clinicians^10^, the prediction model was developed into an Asthma Diagnosis Decision Aid (ADxDA), a CDSS for use in UK primary care. The aim of this study was to explore the feasibility of deploying the CDSS in UK primary care and acceptability of the CDSS to clinical users to inform the potential for future development and evaluation of effectiveness.

## Methods

### Study design, ethics, and reporting guidelines

We conducted an observational mixed methods feasibility study with ethical approval from South West—Cornwall and Plymouth Research Ethics Committee (ref: 21/SW/0080). All healthcare professional participants provided written informed consent. Usage data comprised anonymous summary statistics; no patient identifiable data were collected. The Consolidated Criteria for Reporting Qualitative Research (COREQ) guided reporting (Table S1)^11^.

### Intervention

#### Prediction model

The intervention included an asthma diagnosis CDSS (known as ADxDA), which operationalised a prediction model and accompanying patient website with information about asthma diagnosis. Development and validation of the asthma diagnosis prediction model has been reported previously^8^. In brief, logistic regression was used to derive a prediction model to support primary care clinicians assess the probability of an asthma diagnosis in children and young people (aged 5-25 years). Following internal validation, the final model was externally validated in routinely collected electronic health records (EHR). Predictors in the final model were wheeze, cough, breathlessness, hay-fever, eczema, food allergy, childhood exposure to cigarette smoke, prescription of a short acting beta agonist and the past recording of lung function/reversibility testing.

#### CDSS design

The CDSS was designed with input from stakeholders (specifically individuals with asthma, parents of children with asthma, general practitioners (GP), nurses and a respiratory paediatrician) through two qualitative studies and user experience workshops^9,10^. Based on stakeholder advice, the CDSS was installed directly on GP computers which enabled specific coded information to be pulled directly from the EHR. The CDSS automatically retrieved the patient’s name, date of birth, and coded data required for the prediction model, and supported health professionals to weigh up the probability of asthma and consider the most appropriate next steps to confirm or refute a diagnosis.

**Fig. 1:**
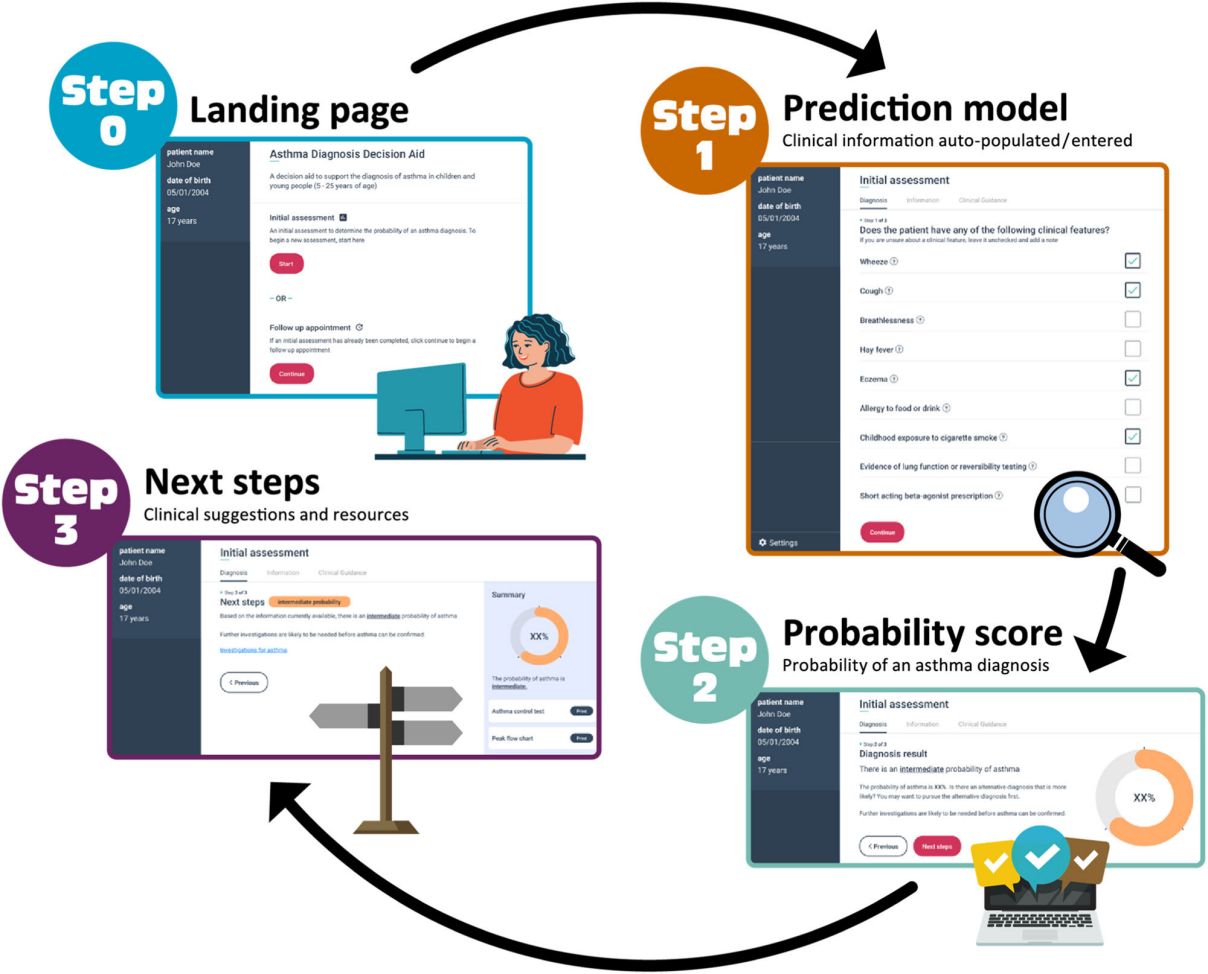
Screenshots from the asthma diagnosis clinical decision support system. Step 0: Landing page. Step 1: information required by the prediction model was imported directly from the electronic health records and could be modified or supplemented by the clinical user. Step 2: The probability of asthma diagnosis, calculated by the prediction model was presented. Step 3: Recommended next steps tailored to the probability of asthma diagnosis (low, intermediate, or high probability) were provided.

The CDSS guided clinical users through three steps: (1) entering information required by the prediction model; (2) visualising the probability of an asthma diagnosis based on the output of the prediction model; 3) considering recommended next steps based on the probability of asthma diagnosis (Fig. 1, Figs. S1–S5). Recommendations were adapted from the British Thoracic Society/Scottish Intercollegiate Guideline Network (BTS/SIGN) and The National Institute for Health and Care Excellence (NICE) asthma guidelines^12,13^, to ensure relevance for health professionals practicing across the UK. The CDSS also contained clinical guidance on how to follow up patients at a subsequent appointment (including interpreting investigations, starting treatment and supporting self-management), and information about how the CDSS was developed. A ‘copy-to-clipboard’ function allowed information from the CDSS (the time and date of use, selected predictors, and the calculated probability score) to be copied and pasted into the patient’s EHR. The CDSS was not automatically triggered, so users were required to consider asthma as a possible differential diagnosis and open the software.

#### Patient website

Following stakeholder advice^9^, we developed an accompanying website providing general information about asthma and the process of making a diagnosis.

### Setting, population and procedure

#### Setting

Supported by clinical research networks (CRNs), the study was advertised to GP practices in England (East Region, Kent Surrey and Sussex, Middlesex, Yorkshire/Humberside) and Scotland (NHS Greater Glasgow and Clyde, NHS Highland, NHS Lothian, NHS Lanarkshire). Upon receipt of an expression of interest, LD, ED or AC met the practice team to discuss the study. Recruited practices were sent a study pack and confirmed participation in writing. Achieving governance approval took up to nine months in some regions, which meant installation of the software was delayed for some recruited practices. Each practice received written instructions and support to install and setup the CDSS and were offered a training session on using the CDSS. Practices used the CDSS for up to six months, after which the software was uninstalled.

#### Population and practice procedure

The study population consisted of GPs and nurses who worked at a participating practice and used the CDSS. Participants were asked (but not incentivised or reminded) to use the CDSS during consultations with children and young people who presented with respiratory symptoms, which may have been due to asthma. The CDSS prompted clinician-users to gain verbal permission from the patients/parents to use the CDSS during consultation.

### Data collection and analysis

#### Quantitative data

The following CDSS usage data were collected: CDSS opened (Step 0), probability calculated (Step 1 and 2), diagnostic recommendations page opened (Step 3), copy-to-clipboard function used, follow-up section opened. No patient data were collected. Users and practices were anonymised. Data were held in accordance with European Union General Data Protection Regulation (GDPR)^14^ and health board/regional governance requirements. Usage data were presented as counts, stratified by total number of events and number of users.

#### Qualitative data

Qualitative interviews provided insight into the feasibility of deploying the CDSS in UK primary care and acceptability of the CDSS from clinician-user’s perspective. GPs and nurses at participating practices who had used the CDSS were invited by email to take part in individual semi-structured interviews. Practices were re-imbursed for the interviews at standard rates. We aimed to recruit participants with a range of clinical roles, years of general clinical/asthma experience, and diverse practice populations, with at least one health professional from each practice. After receipt of an expression of interest and signed consent form, interviews were conducted by telephone using a pre-specified topic guide^15^. Topic guides (Fig. S6) were developed and piloted with a professional advisory group, and members of the Asthma UK Centre for Applied Research Patient and Public Involvement (PPI) group.

Interviews were conducted between 16 August 2022 and 24 November 2022 by an experienced qualitative health services researcher (A.C.). Interviews were audio-recorded, transcribed verbatim and anonymised. Sampling continued in an iterative manner with analysis until theoretical saturation was achieved, i.e. until no new views or experiences were identified. Transcripts were uploaded to QSR NVivo 12 software for coding and interpretation^16^. Data from semi-structured interviews were analysed thematically. This approach enabled us iteratively to develop an understanding of the meanings, views and lived reality of using the CDSS^17,18^. A second researcher (L.D.) independently checked transcripts and themes. Any areas of conflict were resolved by a third researcher (H.P.) and through regular team meetings.

## Results

**Fig. 2:**
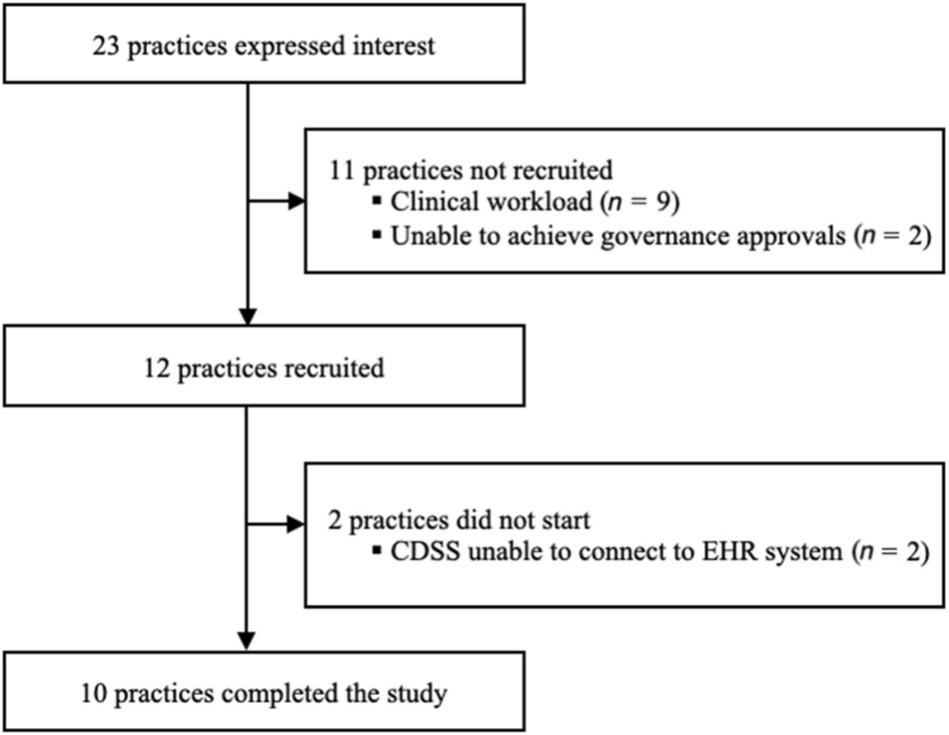
Study flowchart.

Expressions of interest were received from 23 practices from six regions. Of the practices who expressed interest, 11 did not start the study due to clinical workload or governance approvals not realised in time (Fig. 2). Two practices were recruited but did not start the study as the CDSS was unable to connect to the computer system they used (EMIS Web). Consequently, 10 practices from two regions (NHS Greater Glasgow and Clyde, NHS Lothian) were recruited and completed the study. The first practice started on 23 March 2022. Data collection finished on the 30 November 2022.

### Quantitative results

During the study period, the CDSS was used by 75 out of a possible 94 clinicians in recruited practices with the software opened 313 times. The median engagement time per user was 1.8 min (interquartile range 1.2 to 3.5). The probability of asthma diagnosis was calculated 165 times (Table 1).

**Table 1:** Usage data for the CDSS.

Event	Event description	Number of users	Number of events
Session started	CDSS opened	75	313
Probability calculated	Probability score calculated	25	165
Diagnosis page read	Diagnostic recommendations opened	25	122
Copy to clipboard	CDSS output copied to the clipboard	20	89

### Qualitative results

11 participants were recruited from eight GP practices. Table 2 shows practice and participant characteristics. Interviews lasted 14–38 min with a mean duration of 24 min. From these data, four themes emerged: (1) Experiences using the CDSS; (2) CDSS features and acceptability for clinical practice; (3) Impact of using the CDSS; and (4) Involving patients in the consultation. Participant ID, clinical role and years of general clinical experience are reported after each quote.

**Table 2:** Practice and participant characteristics.

Practice ID	Practice population	Patients in the 15% most deprived areas^*^	Practice Urban/ Rural category^†^	Participant ID	Clinical role	Sex	General clinical experience (years)	Asthma specific experience (years)
1	6500	0%	2	C33	NP	Female	5	NA
				C34	GP	Male	19	NA
2	11,000	25%	1	C38	GP	Male	17	17
				C40	ANP	Female	4	2
3	10,400	5%	1	C42	ANP	Female	10	2
4	9100	5%	1	C35	NP	Female	6	1
5	12,600	0%	3	C36	GP	Female	4	2
6	12,300	10%	2	C37	ANP	Female	17	6
7	6700	15%	1	C32	ANP	Female	15	9
				C41	GP	Male	2	NA
8	1400	40%	1	C39	NP	Female	26	7
9	5900	50%	1	-				
10	10,000	5%	1	-				

GP practice data from Public Health Scotland, General Practice - GP workforce and practice list sizes, 2022. Available from: https://publichealthscotland.scot/publications/general-practice-gp-workforce-and-practice-list-sizes/general-practice-gp-workforce-and-practice-list-sizes-2012-2022/.

*Percentage of practice patients in the 15% most deprived areas of Scotland.

^†^Urban/Rural categories: 1 = Large Urban Areas, 2 = Other Urban Areas, 3 = Accessible Small Towns, 4 = Remote Small Towns, 5 = Very Remote Small Towns, 6 = Accessible Rural, 7 = Remote Rural, 8 = Very Remote Rural.

*GP* general practitioner, *NP* nurse practitioner, *ANP* advance nurse practitioner.

#### Theme 1: Experiences using the CDSS

##### Installation and preparation for use

Participants were generally satisfied with the training received prior to use, though some would have preferred additional support:

I think I was well prepared. It’s [the CDSS] not complicated; the interface itself is quite apparent and easy to interact with, so to navigate around the actual software is not difficult. (C34, GP, 19 years)

Of those who felt dissatisfied, one participant would have preferred written rather than digital instructions. Another participant experienced a delay between training and CDSS installation, making them less confident in using the software:

When it actually came to it being live and on my screen and able to start using it, a lot of it was trial and error and I’m thinking, am I using it right, am I not using it right? (C40, Advanced Nurse Practitioner (ANP), 4 years)

Participant C42 felt she would have benefited from additional monitoring to check she was using the CDSS correctly:

The only other thing would be maybe if, once we’d been using it, we had somebody to review our usage of it. […] So, I think going into it, we used it and then I wasn’t sure if I was using it right, you know? (C42, ANP, 10 years)

##### Ease of use

Clinicians generally found the CDSS user-friendly, and straightforward to use compared to other clinical tools they used:

I think the user interface is quite nice, I think it’s very easy to use, in terms of just clicking the symptoms that were appropriate. (C41, GP, 2 years)

##### Compatibility with existing software

The compatibility of the CDSS with the primary care EHR software (Vision and EMIS PCS) was acceptable, but not all desired features were available. The intention was that when connected to the patient EHR, relevant clinical details would automatically populate the CDSS, a feature which was appreciated by participants for whom it worked, and *‘would have been a better experience’* for those in practices where this was not possible:

It was great that it pulled out the relevant history […] I hadn’t twigged that the patient had salbutamol in the past and it [the CDSS] did bring that up. (C38, GP, 17 years)

Whilst the CDSS extracted existing coded data, it was not able to write clinical codes into the EHR. Instead, a *copy-and-paste* function was used as a workaround to enable information from the CDSS to be recorded as free text:

Well, if I was using the tool and I ticked all the boxes and it said, high probability, it would be good if that just went into the patient’s notes. (C40, ANP, 4 years)

#### Theme 2: CDSS features and acceptability for clinical practice

##### Probability score

Participants were generally positive about the calculation and presentation of the probability of asthma diagnosis:

I like the wee circular chart that shows you [the probability]. I like that. I liked how easy it is to use as well, actually. Like, you just tick the boxes, and it just works it out for you. Which is quite nice. (C37, ANP, 17 years)

In contrast, one participant was frustrated that most patients were categorised into the intermediate group instead of providing a definitive diagnosis.

You need to say, well, this is most likely, high likely, or not likely at all, rather than most people coming up in the middle, which is not really helpful, because that’s kind of why you…[laugh] that’s why you want to use the tool. (C38, GP, 17 years)

##### Barriers for using the CDSS

The barriers reported by participants included difficulty connecting to the EHR, and a lack of detail in the clinical features asked about:

Just to show [the presence or absence of] a cough as a tick, I wouldn’t know if that was a cough that was associated with a cold the day before or the week before or every month for the last year (C40, ANP, 4 years)

In addition, a limited number of suitable cases with which to use the CDSS and not being prompted to use the CDSS were identified as barriers:

I’m just trying to think, I had a patient that I probably could have used it, and I didn’t and I was like “Ah, I forgot to”, and I don’t know why I didn’t. (C42, ANP, 10 years)

##### Use in routine clinical practice

Participants had a range of views about whether they would use the CDSS in their everyday practice. Some participants were enthusiastic:

I think it’s a good tool, I would definitely use it on the right patients. I would have no problem with using it at all, and I think it just adds an extra layer to the consultation, as well. (C41, GP, 2 years)

However, others were doubtful it would add to their existing practice:

For myself it would only be to find out the probability. I think because when it tells you the next steps and the next investigations, an asthma nurse will already know what they are anyway (C33, NP, 5 years)

##### Users most likely to benefit

There were contrasting views about who would benefit most from using the CDSS. Most participants considered the CDSS would be helpful to newly qualified staff, trainees, or those less experienced in assessing undiagnosed respiratory symptoms. C34, an experienced GP with an interest in clinical education explained:

I think younger colleagues and certainly GP trainees would find it really helpful and I think it would educate, which I think is really helpful as well, I mean as an educational tool it’d be good’. (C34, GP, 19 years)

Another GP, C36, added that experienced GPs would probably not need the CDSS:

I think most of my peers would, kind of, have enough experience with this that they would either not think it’s asthma or give them a trial of an inhaler. (C36, GP, 4 years)

Interestingly, whilst GPs tended to feel that nurses might benefit from using the CDSS, nurse participants suggested GPs (particularly those with less experience) would benefit from the CDSS because they see patients presenting with symptoms that could be asthma:

I would say GPs in general, definitely. Those who are probably not experienced in asthma diagnosis. So, I would say those are the people that would benefit. (C32, ANP, 15 years)

An alternative view, was that the CDSS might appeal to those with a specialist interest in asthma:

It [the CDSS] would probably appeal to some that … maybe have an interest in respiratory, so those doctors or ANPs that have an interest in that group. (C35, NP, 6 years)

Aside from clinical experience, one participant suggested that users with an interest and experience in using technology might be more likely to use the CDSS:

If there was a GP that was more computer literate and up for doing those sorts of things, I think they would use it and would be quite happy using it. (C39, NP, 26 years)

#### Theme 3: Impact of using the CDSS

Participants described how the CDSS might have an impact on clinical practice.

##### Change of approach

The CDSS could streamline the process and led some, but not all, participants to change aspects of their diagnostic assessment of asthma:

‘I probably did it [the diagnostic assessment] more in front of the patient… [currently] I might phone them back after I’d had a look at the records if I hadn’t had time while I had them visiting or attending. So, I suppose it definitely helped me do it quickly and promptly in front of a patient without hesitation’. (C32, ANP, 15 years)

The same participant also described how ‘*if the percentage had been particularly high, I may have acted more promptly’*.

Having a list of clinical features to ask about acted as a useful reminder for some participants:

Previously the GP would say ‘Oh, have you asked about this and this and this?’ And I’d go ‘Oh no’. I didn’t forget to ask about it because it [the CDSS] was sitting in front of me. (C39, NP, 26 years)

##### Influence of the CDSS on decision making

Participants had mixed views about whether the CDSS influenced their decision making. Having the probability of asthma diagnosis calculated by the CDSS contributed to the decision making of C37:

Because it worked out the overall probability for you …like the percentage and stuff, that would sway me one way or the other. (C37, ANP, 17 years)

Other participants felt the CDSS verified what they already thought. This provided affirmation for one participant:

‘It maybe confirmed what I was going to do but, yes, I don’t think it changed my plan or management, […] it, kind of, reaffirmed what I was thinking rather than changed it’. (C36, GP, 4 years)

Not leading to a change in practice led other participants to *‘stop being as enthusiastic’* (C42, ANP, 10 years) about using the CDSS.

#### Theme 4: Involving patients in the consultation

##### Patient engagement

Most participants found the CDSS supported patient (and/or parents) engagement during a consultation for possible asthma. C41 recalled how seeing the symptoms inputted into the CDSS was valuable:

I think she [the patient] appreciated, you know, kind of seeing the symptoms displayed, and then what that would generate in terms of a probability, I think she did appreciate that. (C41, GP, 2 years)

Other participants found the opportunity to visualise the concept of the probability of asthma helpful:

So, it’s difficult to kind of explain to a patient anyway but the percentage definitely assisted with their kind of understanding. So, I kind of say it’s a likelihood, it’s not a definitive thing. That’s how I described it. (C32, ANP, 15 years)

Displaying the probability of asthma could also be useful to help explain why asthma was less likely:

Sometimes a parent might come in very fixed on it being asthma. If you maybe get to a point using the tool saying, well, actually, you know, if we look at this, it’s maybe not that. Kind of reassures you and the parent a bit more that it wasn’t just all coming from one person or just from me or from no testing, really. (C36, GP, 4 years)

##### Computer screen sharing

Participants were mixed in their views and use of screen sharing. Four participants did share their screen and found the CDSS useful for communicating aspects of the diagnostic assessment with patients:

And I think it would probably be good for them to see when I click on investigations for asthma and when I explain to them what the peak flow monitoring does. If they see that up on the screen as further investigations, then that would maybe just reassure them a wee bit more. (C33, NP, 5 years)

In contrast, one participant did not feel screen sharing would be useful:

I don’t hide it from them. I don’t think it’s particularly helpful for the patients to see so I don’t think it’s been designed for that. […] So no, I haven’t, but not for…just because I don’t think it would be helpful actually. (C34, GP, 19 years)

## Discussion

### Main findings

During this feasibility study, the CDSS was installed in 10 practices and used by 75 health professionals. The CDSS was acceptable to the participants who used it, who described the ease of use, auto-population of information from the EHR, the visual design and presentation of the probability score positively. However, engagement with the CDSS was less than anticipated, which may reflect the feeling that the software would not substantially help them in their clinical practice.

### Strengths and limitations

Strengths of the study included the use of mixed methods which enabled us to evaluate usage of the CDSS together with responses from in depth participant interviews. Recruiting a range of practices based on practice size and socio-economic status enabled the CDSS to be tested in different settings. Having a non-clinical background enabled AC to ask participants more enquiring questions about clinical aspects and be less restricted when asking about the CDSS. The influence of any single individual in the interpretation of the results was minimised by involving multiple team members, including a steering group.

Our study also had limitations. Achieving governance approval was delayed in some regions. Consequently, there was a long period between training and installation at some sites which affected participant experience and may have reduced the number of clinician users. The quantitative data collection did not allow us to identify the probability scores or final diagnosis of individual patients. The identity of clinician users was hidden, so it was not possible to compare how the CDSS was used by participants who contributed an interview compared to those not interviewed. For the qualitative analysis, we received fewer expressions of interest than anticipated which meant every participant that expressed interest was recruited and interviewed although we achieved a spread of age/experience, and the gender imbalance reflects the predominantly female practice nurse workforce. Participants who took part in interviews may have been more enthusiastic about using the CDSS than those who did not express interest, which may have introduced bias in the sample, and contributed to data saturation being achieved despite a relatively small sample.

### Interpretation

Systematic review evidence has highlighted common barriers for adopting CDSS into clinical practice which include design and technical concerns, lack of training, doubt about relevance or scientific quality, time constraints, problems with interoperability, lack of agreement with the applicability to the clinical situation and challenge to clinician autonomy^19^. In our study, participants were generally positive about interoperability, though the inability of the CDSS to write code into the EHR after use was a frustration for some and a minority were unable to connect to the EHR. As adoption of new technology in routine clinical practice is often provided with minimal training, in this feasibility study we opted not to provide extensive training or closely monitor use. However, with delays between training and installation, and the lack of an automated prompt, CDSS usage was less than anticipated, which could have been improved if we had provided greater support for users. In addition, the relevance of the CDSS was questioned by some participants, who expected the software to provide clearer direction (i.e. asthma yes or no) and more tailored recommendations.

Based on the same systematic review, the main facilitators for health professionals choosing to use a CDSS were perceived usefulness, ease of use, and impact on clinical uncertainty^19^. In this sample, which included participants with 2–19 years of experience, the CDSS was felt to be most useful for trainees or inexperienced clinicians. Whilst clinical experience alone does not ensure that a health professional is a good diagnostician^20^, the opportunity to gain familiarity with a variety of clinical cases and refine approaches for clinical reasoning is thought to help^21^. Thus, for professionals who have experience of making an asthma diagnosis, it is understandable that the perceived usefulness of the CDSS was less clear, though we have no means of knowing if this confidence is misplaced. Using a validated prediction model^8^, the CDSS calculated the probability of an asthma diagnosis. In keeping with asthma guidelines^12^, recommendations in the CDSS were to complete further testing (if intermediate or high probability) or investigate an alternative diagnosis (if low probability). Therefore, whilst calculating the probability was novel and potentially reduced clinical uncertainty, experienced clinicians were still left with the same clinical scenario and having to decide which investigation to try or whether to attempt a treatment trial.

In general, participant’s feedback gave a sense that the CDSS was ‘nice, but more useful for someone else’. GPs felt that nurses would benefit more because they tended to see patients with asthma. Some nurses felt that GPs would be more likely to benefit as assessing children/young people with undiagnosed symptoms was not something for which they were responsible. These findings were similar to previous qualitative work^10,22^ and reflects the current approach to asthma provision in UK primary care, which in many practices is largely nurse led^23^. Other factors may be differences in the training received and the differing clinical roles of doctors and nurses.

Were the CDSS further developed and evaluated (for example, in a trial), it would be necessary to extract additional data from the EHR to understand the clinical decision making and outcome of the diagnostic assessment. For example, an automated prompt alerting health professionals to possible cases of asthma could increase CDSS usage and enable its effect on asthma diagnosis to be better understood. Gaining the views of children, young people and parents toward the CDSS and if it influenced their attitude toward the resulting diagnosis and decision to take ongoing treatment could suggest another possible benefit.

### Implications for research and practice

The CDSS requires further development before being available in routine clinical practice. For researchers, further evaluation of the prediction model for asthma diagnosis could enable more nuanced clinical details (such as the character, duration or variability of cough) to be considered as predictors, the possibility for the probability score to provide a definitive clinical recommendation (i.e. asthma or not, and optimal investigation to enhance diagnostic accuracy) and the external validity of the model to be assessed in other settings. Understanding how to prompt clinicians without being an irritation is important, especially in the context of respiratory symptoms that are very common in children/young people (cough, ‘chesty’) or used inconsistently (‘wheeze’^24^ where only a small minority have asthma. Making a diagnosis of asthma remains challenging particularly when access to objective tests is limited. For clinicians supporting patients facing long delays before a diagnosis can be achieved, using visual aids to communicate probability and promote engagement with the diagnostic process was an aspect of the CDSS that was well received and helped to engage patients.

## Conclusion

In this feasibility study, an asthma diagnosis CDSS was acceptable to primary care health professionals who used it and was felt to be particularly helpful for trainees and clinicians with less experience in diagnosing asthma.

## Supplementary information

ADxDA_FeasibilityPaper_SupplementaryMaterial

## Data Availability

The datasets generated and analysed during the current study are not publicly available due to privacy or ethical restrictions but may be available from the corresponding author on reasonable request.
