# Supplementary material for: Use and acceptability of an asthma diagnosis clinical decision support system for primary care clinicians: an observational mixed methods study
Source: NPJ Prim Care Respir Med. 2024 Nov 27;34:40. doi: 10.1038/s41533-024-00401-x (PMC11603350; doi:10.1038/s41533-024-00401-x)
Supplement: Supplementary file 1 — ADxDA_FeasibilityPaper_SupplementaryMaterial [file 41533_2024_401_MOESM1_ESM.docx]

**Use and acceptability of an asthma diagnosis clinical decision support system for primary care clinicians: an observational mixed methods study**

**Supplementary Material**

[Table S1: COREQ (COnsolidated criteria for REporting Qualitative research) Checklist 2](#_Toc177972579)

[Figure S1: CDSS Step 0 – Landing page 4](#_Toc177972580)

[Figure S2: CDSS Step 1 – Prediction model 5](#_Toc177972581)

[Figure S3: CDSS Step 2 – Probability Score 6](#_Toc177972582)

[Figure S4: CDSS Step 3 – Next Steps 6](#_Toc177972583)

[Figure S5: CDSS – Follow up appointment 7](#_Toc177972584)

[Figure S6: Healthcare Professional Topic Guide 8](#_Toc177972585)

## Table S1: COREQ (COnsolidated criteria for REporting Qualitative research) Checklist

| **Topic** | **Item No.** | **Guide Questions/Description** | **Page No.** |
| --- | --- | --- | --- |
| **Domain 1: Research team and reﬂexivity** | | | |
| *Personal characteristics* | | | |
| Interviewer/facilitator | 1 | Which author/s conducted the interview or focus group? | 7 |
| Credentials | 2 | What were the researcher’s credentials? E.g. PhD, MD | 7 |
| Occupation | 3 | What was their occupation at the time of the study? | 7 |
| Gender | 4 | Was the researcher male or female? | - |
| Experience and training | 5 | What experience or training did the researcher have? | 7 |
| *Relationship with participants* | | | |
| Relationship established | 6 | Was a relationship established prior to study commencement? | - |
| Participant knowledge of  the interviewer | 7 | What did the participants know about the researcher? e.g. personal goals, reasons for doing the research | - |
| Interviewer characteristics | 8 | What characteristics were reported about the interviewer/facilitator?  e.g. Bias, assumptions, reasons and interests in the research topic | 7 |
| **Domain 2: Study design** | | | |
| *Theoretical framework* | | | |
| Methodological orientation and Theory | 9 | What methodological orientation was stated to underpin the study? | 7 |
| *Participant selection* | | | |
| Sampling | 10 | How were participants selected? | 6 |
| Method of approach | 11 | How were participants approached? e.g. telephone, mail, Email | 6 |
| Sample size | 12 | How many participants were in the study? | 7 |
| Non-participation | 13 | How many people refused to participate or dropped out? Reasons? | 7 |
| *Setting* | | | |
| Setting of data collection | 14 | Where was the data collected? e.g. home, clinic, workplace | 6 |
| Presence of non-  participants | 15 | Was anyone else present besides the participants and researchers? | - |
| Description of sample | 16 | What are the important characteristics of the sample? e.g. demographic data, date | 10 |
| *Data collection* | | | |
| Interview guide | 17 | Were questions, prompts, guides provided by the authors? Was it pilot tested? | 6 |
| Repeat interviews | 18 | Were repeat interviews carried out? If yes, how many? | - |
| Audio/visual recording | 19 | Did the research use audio or visual recording to collect the data? | 6 |
| Field notes | 20 | Were ﬁeld notes made during and/or after the interview or focus group? | - |
| Duration | 21 | What was the duration of the interviews or focus group? | 7 |
| Data saturation | 22 | Was data saturation discussed? | 7,16 |
| Transcripts returned | 23 | Were transcripts returned to participants for comment and/or correction? | - |
| **Domain 3: analysis and ﬁndings** | | | |
| *Data analysis* | | | |
| Number of data coders | 24 | How many data coders coded the data? | 7 |
| Description of the coding  tree | 25 | Did authors provide a description of the coding tree? | - |
| Derivation of themes | 26 | Were themes identiﬁed in advance or derived from the data? | 7 |
| Software | 27 | What software, if applicable, was used to manage the data? | 7 |
| Participant checking | 28 | Did participants provide feedback on the ﬁndings? | - |
| Quotations presented | 29 | Were participant quotations presented to illustrate the themes/ﬁndings? Was each quotation identiﬁed? | 8-14 |
| Data and ﬁndings consistent | 30 | Was there consistency between the data presented and the ﬁndings? | 15-18 |
| Clarity of major themes | 31 | Were major themes clearly presented in the ﬁndings? | 8-14 |
| Clarity of minor themes | 32 | Is there a description of diverse cases or discussion of minor themes? | 14 |
| Developed from: Tong A, Sainsbury P, Craig J. Consolidated criteria for reporting qualitative research (COREQ): a 32-item checklist for interviews and focus groups. *International Journal for Quality in Health Care*. 2007. Volume 19, Number 6: pp. 349 – 357 | | | |

## Figure S1: CDSS Step 0 – Landing page


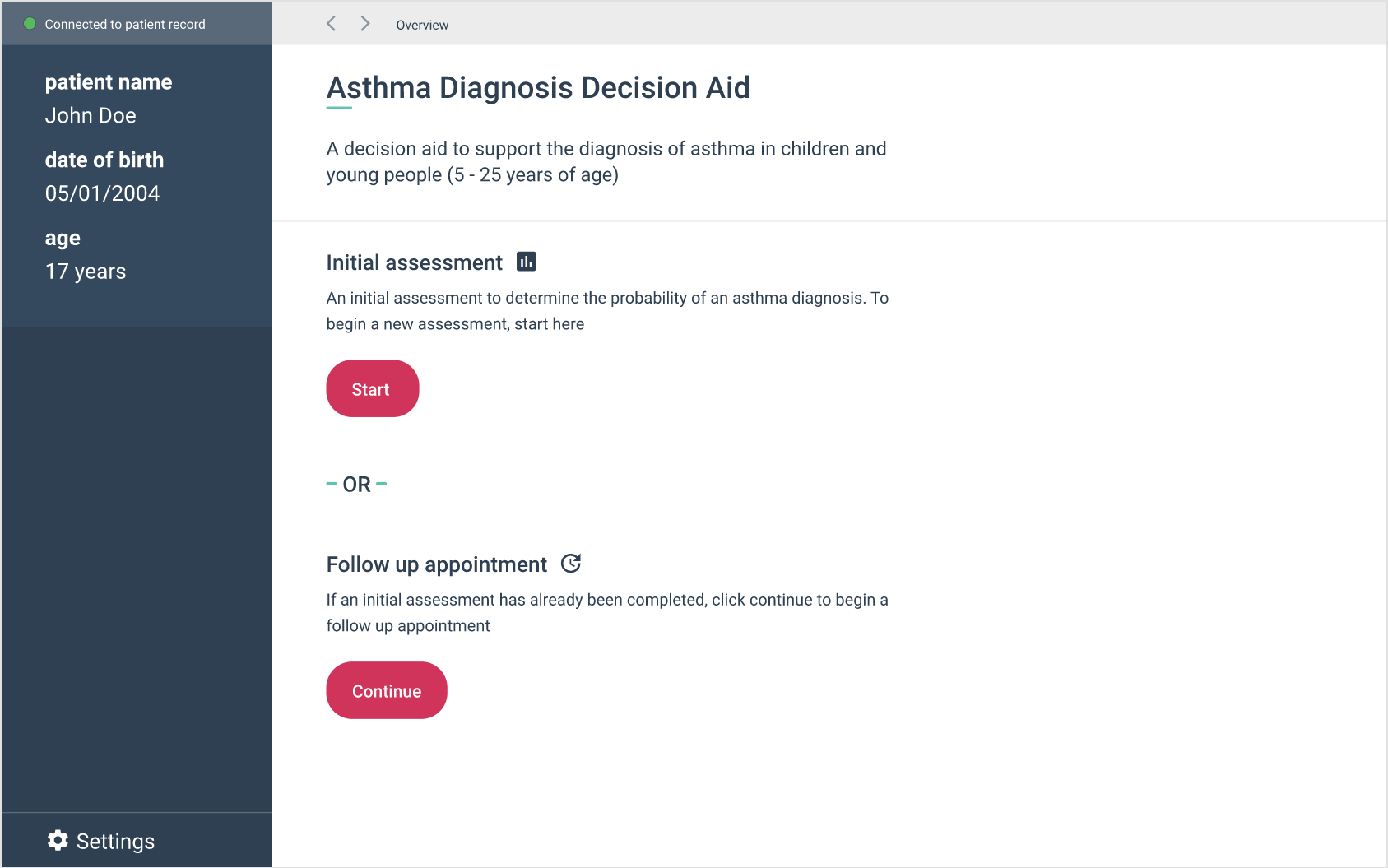


## Figure S2: CDSS Step 1 – Prediction model


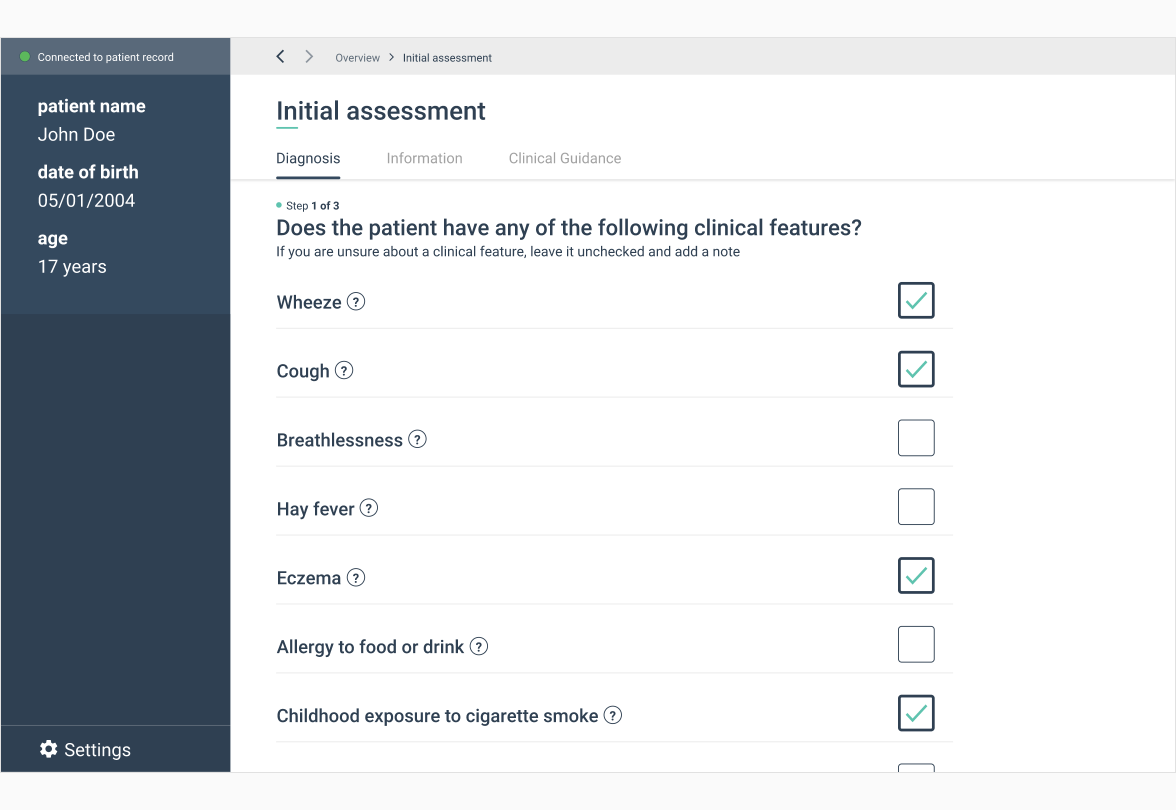


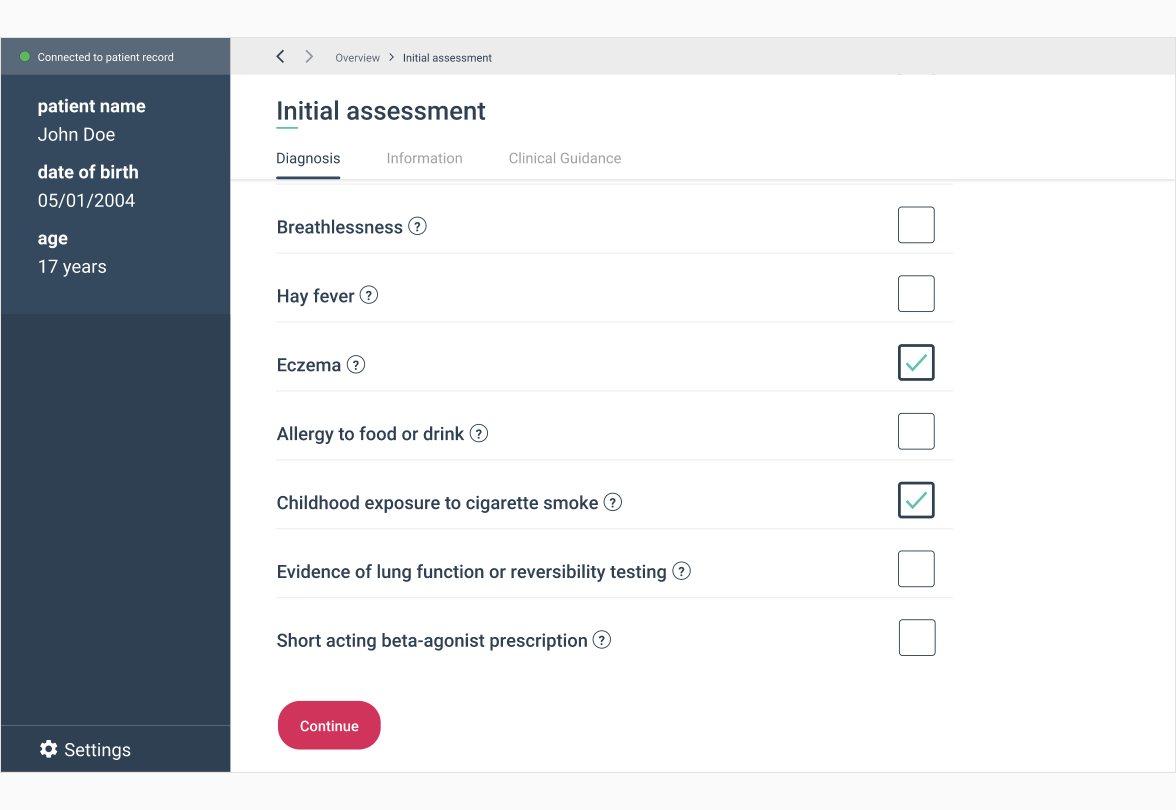


## Figure S3: CDSS Step 2 – Probability Score


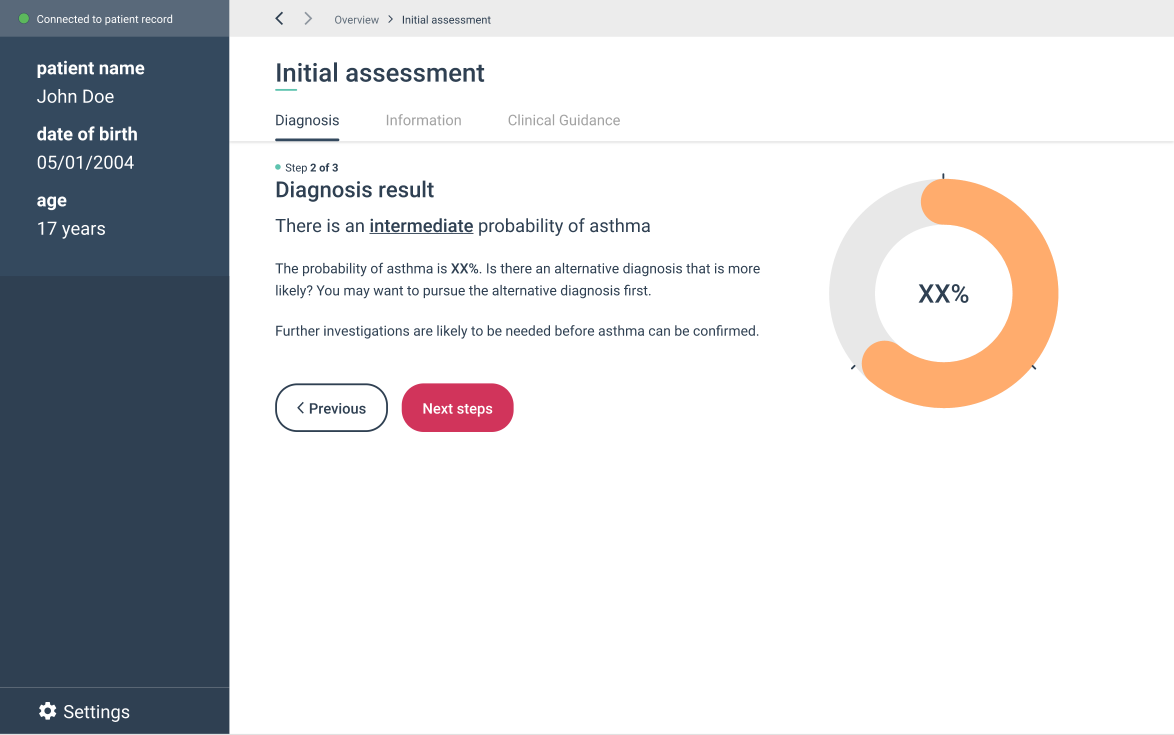


## Figure S4: CDSS Step 3 – Next Steps


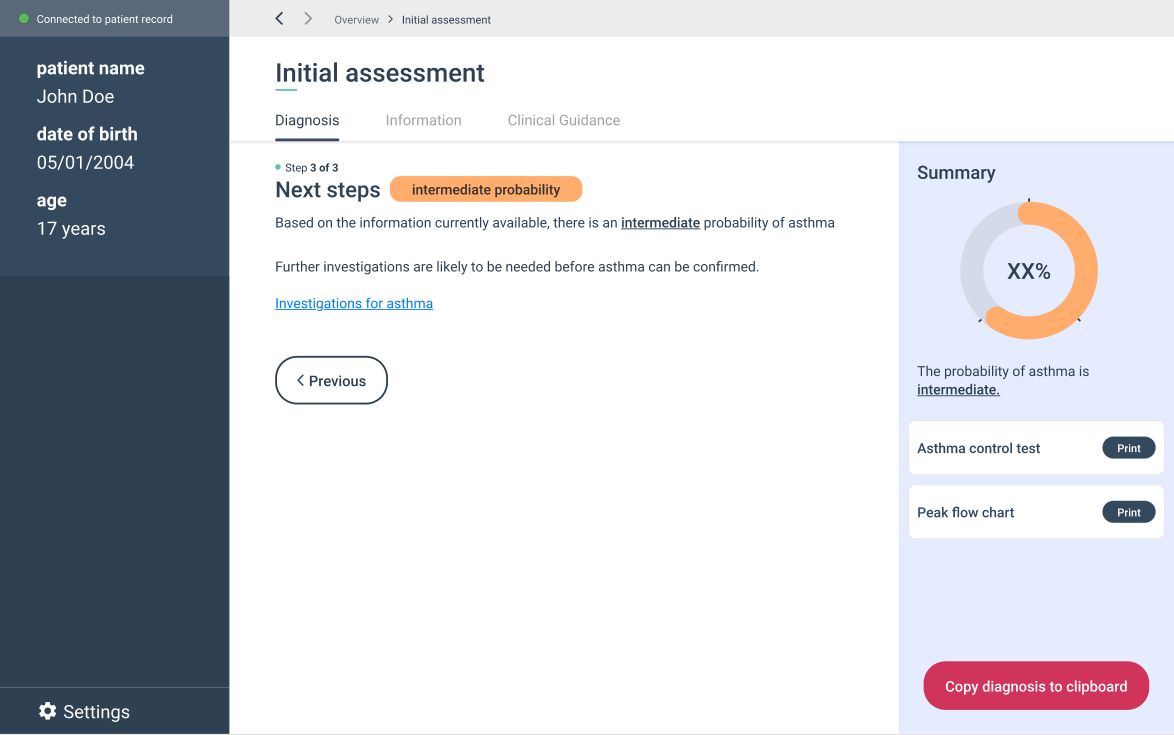


## Figure S5: CDSS – Follow up appointment


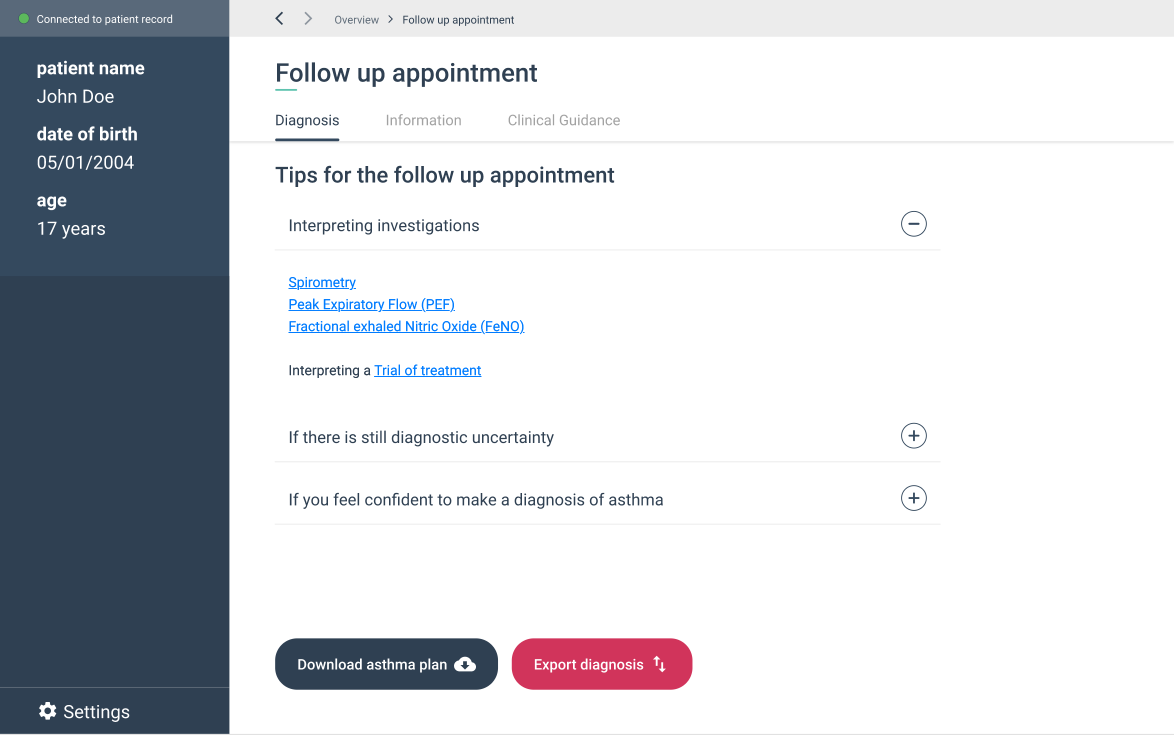


## Figure S6: Healthcare Professional Topic Guide
